# Correction: A graded neonatal mouse model of necrotizing enterocolitis demonstrates that mild enterocolitis is sufficient to activate microglia and increase cerebral cytokine expression

**DOI:** 10.1371/journal.pone.0359857

**Published:** 2026-10-01

**Authors:** Cuilee Sha, Trevor Van Brunt, Jacob Kudria, Donna Schmidt, Alisa Yurovsky, Jela Bandovic, Michael Giarrizzo, Joyce Lin, Styliani-Anna Tsirka, Agnieszka B. Bialkowska, Lonnie P. Wollmuth, Esther M. Speer, Helen Hsieh

Sarah Bucher is not included in the author byline. Sarah Bucher should be listed as the ninth author and affiliated with Center for Nervous System Disorders, SUNY-Stony Brook, Stony Brook, New York, United States of America, Department of Surgery, Division of Pediatric Surgery, Stony Brook Medicine. The contributions of this author are as follows: Scrubbed and maintained research data, Conducted a research and investigation process.

Noah Teaney is not included in the author byline. Noah Teaney should be listed as the tenth author and affiliated with Center for Nervous System Disorders, SUNY-Stony Brook, Stony Brook, New York, United States of America, Department of Surgery, Division of Pediatric Surgery, Stony Brook Medicine. The contributions of this author are as follows: Scrubbed and maintained research data, Conducted a research and investigation process.

Amrendra Singh is not included in the author byline. Amrendra Singh should be listed as the 11th author and affiliated with Department of Pediatrics, Division of Neonatology, Stony Brook Medicine, Stony Brook, New York, United States of America. The contributions of this author are as follows: Scrubbed and maintained research data, Conducted a research and investigation process.

There are errors in the Acknowledgement statement. The correct Acknowledgement statement is as follows: The authors would like to thank Drs. Gail Besner, Amalia Napoli, Vincent Yang, Ashwini Phadnis-Moghe, Miguel Maderia, and Yijie Wang for scientific discussion and assistance with this manuscript. The authors would also like to thank Dr. Stella Ku for their contributions to data collection.
