# Supplementary material for: A graded neonatal mouse model of necrotizing enterocolitis demonstrates that mild enterocolitis is sufficient to activate microglia and increase cerebral cytokine expression
Source: PLoS One. 2025 May 30;20(5):e0323626. doi: 10.1371/journal.pone.0323626 (PMC12124527; doi:10.1371/journal.pone.0323626)
Supplement: S1 Fig — Nursing mice gained weight more rapidly compared to formula-fed mice. Nursing mice nearly doubled their weight within 72 hours; two-way ANOVA with Tukey’s post-hoc. Data presented as mean ± standard error of mean (SEM). ****p < 0.0001. Number of mice: nursing, 7; 0% DSS, 40. Because of these differences between nursing and 0% DSS-fed mice, as well as previous findings demonstrating that maternal separation in mice during the first week of life negatively affects neurodevelopment [17], we determined that isolating the mice during feeding is a confounding factor. Therefore, we used 0% DSS fed mice as controls for our experiments using different supplementations of DSS. (PDF) [file pone.0323626.s001.pdf]

Supporting Information

A graded neonatal mouse model of necrotizing enterocolitis demonstrates that mild enterocolitis is sufficient to activate microglia and increase cerebral cytokine expression

Sha, et al.

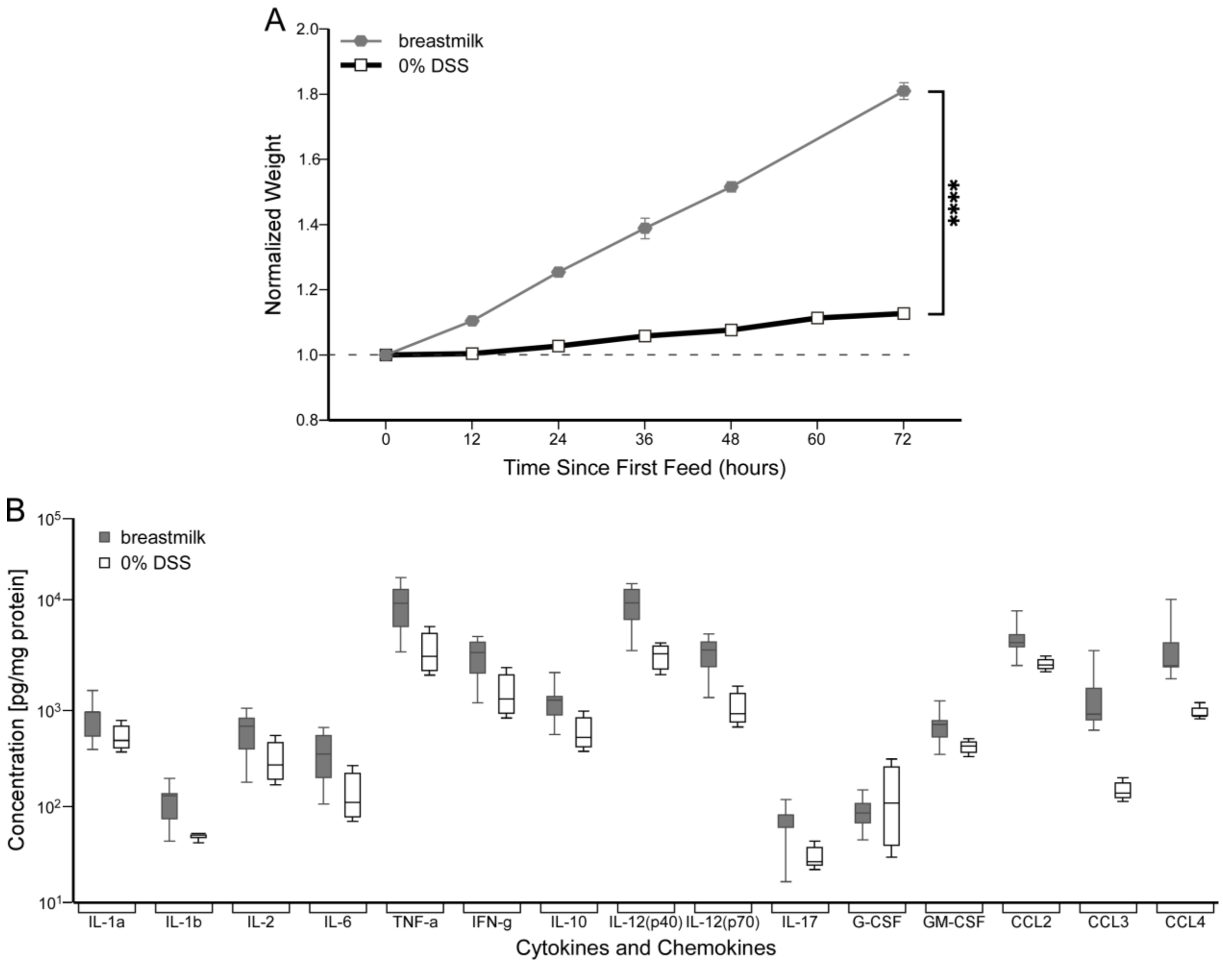

S1 Fig. Nursing mice exhibit notable differences in weight gain and cytokine profile compared to mice fed formula only (0% DSS).

Nursing mice gained weight more rapidly compared to formula-fed mice. Nursing mice nearly doubled their weight within 72 hours; two-way ANOVA with Tukey's post-hoc. Data presented as mean ± standard error of mean (SEM). ****$p < 0.0001$. Number of mice: nursing, 7; 0% DSS, 40.

Because of these differences between nursing and 0% DSS-fed mice, as well as previous findings demonstrating that maternal separation in mice during the first week of life negatively affects neurodevelopment [1], we determined that isolating the mice during feeding is a confounding factor. Therefore, we used 0% DSS fed mice as controls for our experiments using different supplementations of DSS.

1. Nishi M: **Effects of Early-Life Stress on the Brain and Behaviors: Implications of Early Maternal Separation in Rodents.** *Int J Mol Sci* 2020, **21**.
