# Supplementary material for: A graded neonatal mouse model of necrotizing enterocolitis demonstrates that mild enterocolitis is sufficient to activate microglia and increase cerebral cytokine expression
Source: PLoS One. 2025 May 30;20(5):e0323626. doi: 10.1371/journal.pone.0323626 (PMC12124527; doi:10.1371/journal.pone.0323626)
Supplement: S2 Fig — CSS measures were determined using a scoring system from Zani et al. (2008) (18). Data presented for each score component and stratified by feeding condition. (A) Appearance, (B) Natural activity, and (C) Response to touch scores increase over the course of the feeding protocol, even when animals are fed formula alone. Furthermore, higher concentrations of DSS are associated with worse CSS earlier. Two-way ANOVA with Tukey’s post-hoc, p < 0.0001 for all analyses. Data presented as mean ± SEM. Number of mice: 0%, 29; 0.25%, 26; 1%, 26; 2%, 7. (PDF) [file pone.0323626.s002.pdf]

Supporting Information

A graded neonatal mouse model of necrotizing enterocolitis demonstrates that mild enterocolitis is sufficient to activate microglia and increase cerebral cytokine expression

Sha, et al.

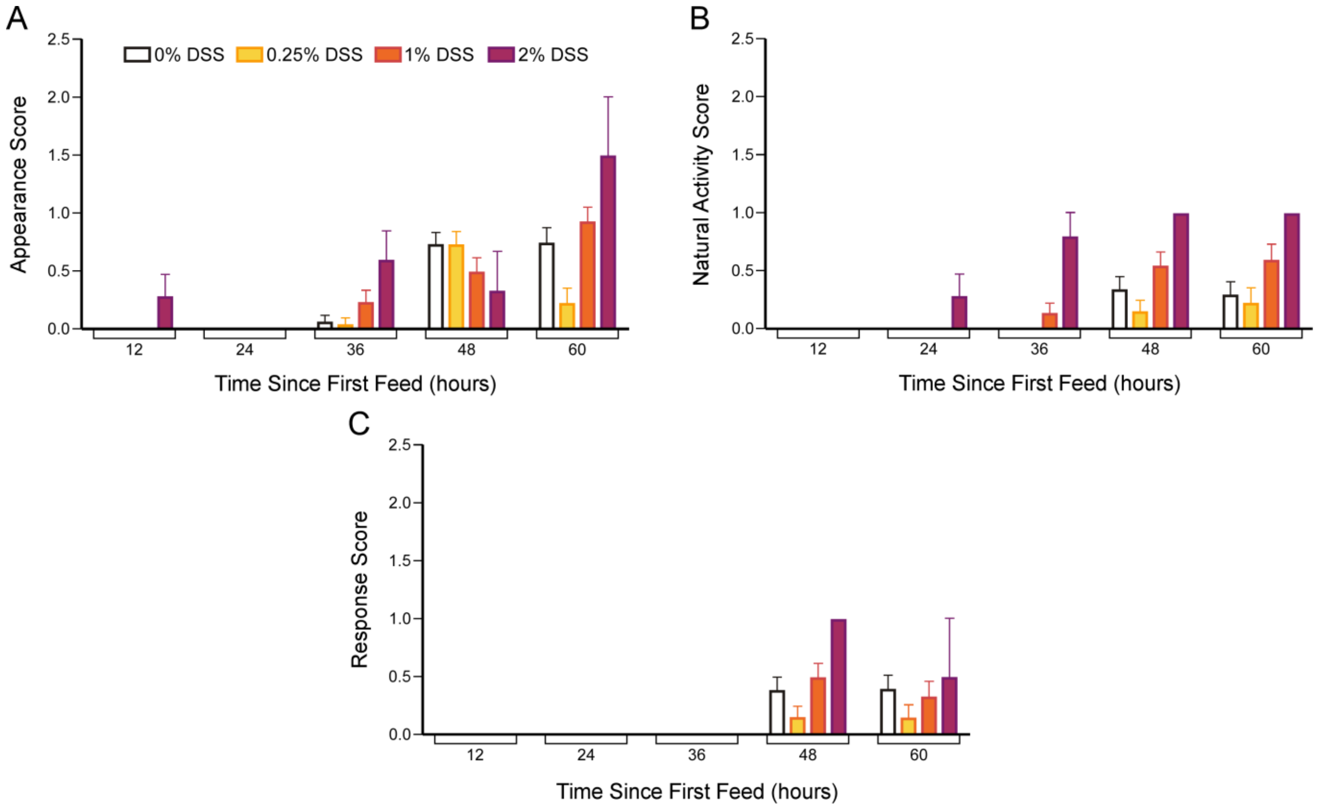

S2 Fig. CSS, in each category, show the same overall trend during feeding as the combined CSS (relates to Fig 1C).

CSS measures were determined using a scoring system from Zani et al. (2008) [2]. Data presented for each score component and stratified by feeding condition. **(A)** Appearance, **(B)** Natural activity, and **(C)** Response to touch scores increase over the course of the feeding protocol, even when animals are fed formula alone. Furthermore, higher concentrations of DSS are associated with worse CSS earlier. Two-way ANOVA with Tukey's post-hoc, $p < 0.0001$ for all analyses. Data presented as mean ± SEM. Number of mice: 0%, 29; 0.25%, 26; 1%, 26; 2%, 7.

2. Zani A, Cordischi L, Cananzi M, De Coppi P, Smith VV, Eaton S, Pierro A: **Assessment of a neonatal rat model of necrotizing enterocolitis.** *Eur J Pediatr Surg* 2008, **18:**423-426.
