# Supplementary material for: A graded neonatal mouse model of necrotizing enterocolitis demonstrates that mild enterocolitis is sufficient to activate microglia and increase cerebral cytokine expression
Source: PLoS One. 2025 May 30;20(5):e0323626. doi: 10.1371/journal.pone.0323626 (PMC12124527; doi:10.1371/journal.pone.0323626)
Supplement: S3 Fig — Intestines were assessed as described in Zani et al (2008) [18]. Bowel (A) consistency, (B) color, and (C) dilation are significantly more severe in mice fed with increasing concentrations of DSS (1% and 2% DSS), compared to lower concentrations of DSS (0% and 0.25% DSS). One-way ANOVA with Tukey’s post-hoc, p < 0.0001 for all analyses. Data presented as mean ± SEM. ***p < 0.001, ****p < 0.0001. Number of mice: 0%, 18; 0.25%, 13; 1%, 18; 2%, 7. (PDF) [file pone.0323626.s003.pdf]

Supporting Information

A graded neonatal mouse model of necrotizing enterocolitis demonstrates that mild enterocolitis is sufficient to activate microglia and increase cerebral cytokine expression

Sha, et al.

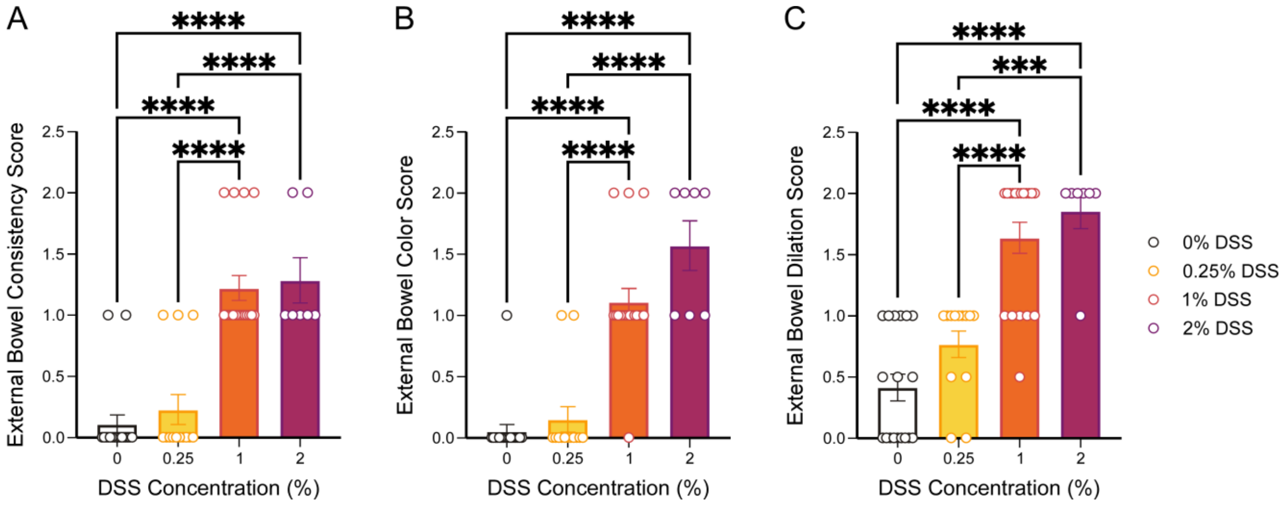

S3 Fig. External bowel scores, by category, show the same overall trend as the combined external bowel score (relates to Fig 2B).

Intestines were assessed as described in Zani et al (2008) [2]. Bowel **(A)** consistency, **(B)** color, and **(C)** dilation are significantly more severe in mice fed with increasing concentrations of DSS (1% and 2% DSS), compared to lower concentrations of DSS (0% and 0.25% DSS). One-way ANOVA with Tukey's post-hoc, $p < 0.0001$ for all analyses. Data presented as mean ± SEM. ***$p < 0.001$, ****$p < 0.0001$. Number of mice: 0%, 18; 0.25%, 13; 1%, 18; 2%, 7.

2. Zani A, Cordischi L, Cananzi M, De Coppi P, Smith VV, Eaton S, Pierro A: **Assessment of a neonatal rat model of necrotizing enterocolitis.** *Eur J Pediatr Surg* 2008, **18:**423-426.
