# Supplementary material for: A graded neonatal mouse model of necrotizing enterocolitis demonstrates that mild enterocolitis is sufficient to activate microglia and increase cerebral cytokine expression
Source: PLoS One. 2025 May 30;20(5):e0323626. doi: 10.1371/journal.pone.0323626 (PMC12124527; doi:10.1371/journal.pone.0323626)
Supplement: S4 Fig — Representative images of the small intestine imaged at 10x then magnified an additional 20x (scale 100 µm). Brown staining represents cleaved capase-3 (CC3), a marker for cellular apoptosis, which is not present in any of the small intestinal crypts. Positive CC3 staining on the tips of the small intestinal villi is not quantifiable because normal conditions also have cellular apoptosis at this location. Number of mice: 0%, 4; 0.25%, 6; 1%, 4; 2%, 4. (PDF) [file pone.0323626.s004.pdf]

Supporting Information

A graded neonatal mouse model of necrotizing enterocolitis demonstrates that mild enterocolitis is sufficient to activate microglia and increase cerebral cytokine expression

Sha, et al.

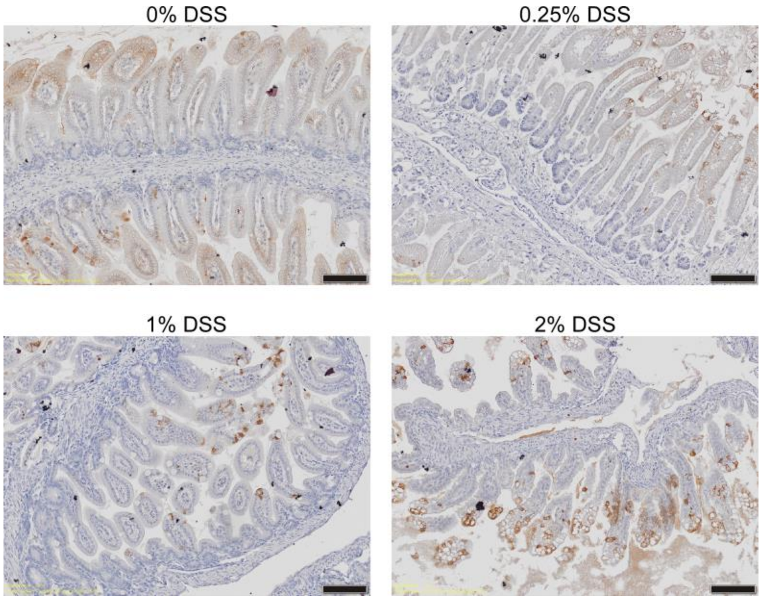

S4 Fig. Cleaved caspase-3 does not appear in the small intestinal crypts across all experimental groups. Representative images of the small intestine imaged at 10x then magnified an additional 20x (scale 100 µm). Brown staining represents cleaved capase-3 (CC3), a marker for cellular apoptosis, which is not present in any of the small intestinal crypts. Positive CC3 staining on the tips of the small intestinal villi is not quantifiable because normal conditions also have cellular apoptosis at this location. Number of mice: 0%, 4; 0.25%, 6; 1%, 4; 2%, 4.
