# Supplementary material for: A graded neonatal mouse model of necrotizing enterocolitis demonstrates that mild enterocolitis is sufficient to activate microglia and increase cerebral cytokine expression
Source: PLoS One. 2025 May 30;20(5):e0323626. doi: 10.1371/journal.pone.0323626 (PMC12124527; doi:10.1371/journal.pone.0323626)
Supplement: S5 Fig — Cytokines/chemokines include (A) IL-1α, p = 0.83, (B) IL-6, p = 0.070, and (C) CCL3, p = 0.054. Simple linear regression with log-transformation of y values was performed. Data presented as boxplots showing min-max. Slope and y intercept with confidence intervals are plotted. ns = not significant (p ≥ 0.05). Number of mice: 0%, 14; 0.25%, 11; 1%, 14; 2%, 4. (PDF) [file pone.0323626.s005.pdf]

Supporting Information

A graded neonatal mouse model of necrotizing enterocolitis demonstrates that mild enterocolitis is sufficient to activate microglia and increase cerebral cytokine expression

Sha, et al.

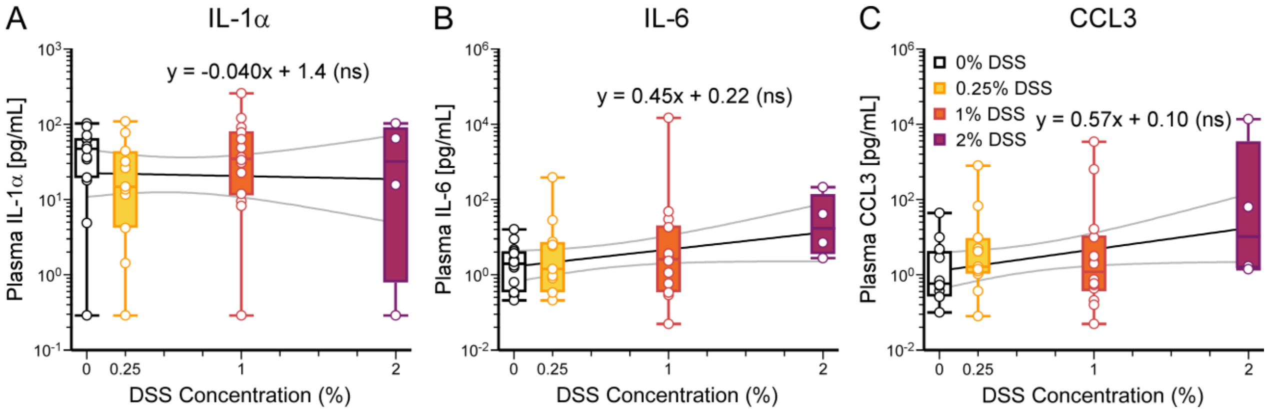

S5 Fig. Some blood plasma cytokine and chemokine concentrations do not significantly correlate with DSS concentration (relates to Fig 5).

Cytokines/chemokines include **(A)** IL-1α, $p = 0.83$, **(B)** IL-6, $p = 0.070$, and **(C)** CCL3, $p = 0.054$. Simple linear regression with log-transformation of y values was performed. Data presented as boxplots showing min-max. Slope and y intercept with confidence intervals are plotted. *ns* = not significant ($p \geq 0.05$). Number of mice: 0%, 14; 0.25%, 11; 1%, 14; 2%, 4.
