# Supplementary material for: A graded neonatal mouse model of necrotizing enterocolitis demonstrates that mild enterocolitis is sufficient to activate microglia and increase cerebral cytokine expression
Source: PLoS One. 2025 May 30;20(5):e0323626. doi: 10.1371/journal.pone.0323626 (PMC12124527; doi:10.1371/journal.pone.0323626)
Supplement: S8 Fig — (A) Representative immunohistochemical images (30x magnification) of the CA1 hippocampal region. Slices are stained blue for DAPI, red for neurons, and green for microglia. Scale bar = 50μm. (B-C) The proportion of (B) neurons, p = 0.046, and (C) microglia, p = 0.018, out of all cells in the CA1 region is comparable across all DSS concentrations. A significant difference was only found when comparing the proportions of neurons between mice fed 1% and 2% DSS, p = 0.027 (S12 Table), and when comparing the proportions of microglia between mice fed 0.25% and 1% DSS, p = 0.018 (S13 Table). One-way ANOVA with Tukey’s post-hoc. Data presented as mean ± SEM. *p < 0.05. n = 9 immunohistochemical images for all experimental groups. (PDF) [file pone.0323626.s008.pdf]

Supporting Information

A graded neonatal mouse model of necrotizing enterocolitis demonstrates that mild enterocolitis is sufficient to activate microglia and increase cerebral cytokine expression

Sha, et al.

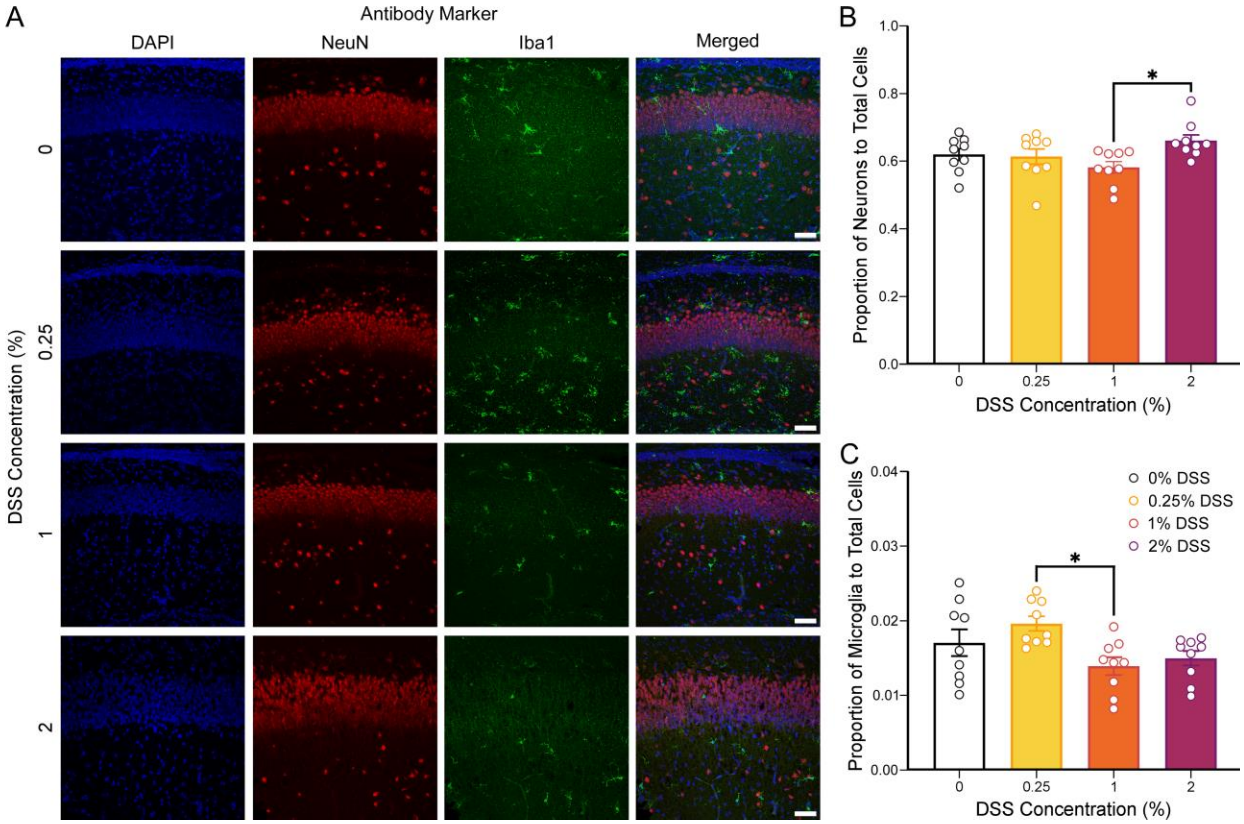

S8 Fig. No significant differences of neuron or microglia proportions in CA1 hippocampal region across all DSS experimental groups.

(A) Representative immunohistochemical images (30x magnification) of the CA1 hippocampal region. Slices are stained blue for DAPI, red for neurons, and green for microglia. Scale bar = 50μm.

(B-C) The proportion of **(B)** neurons, $p = 0.046$, and **(C)** microglia, $p = 0.018$, out of all cells in the CA1 region is comparable across all DSS concentrations. A significant difference was only found when comparing the proportions of neurons between mice fed 1% and 2% DSS, $p = 0.027$ (S12 Table), and when comparing the proportions of microglia between mice fed 0.25% and 1% DSS, $p = 0.018$ (S13 Table). One-way ANOVA with Tukey's post-hoc.

Data presented as mean ± SEM. $*p < 0.05$. n = 9 immunohistochemical images for all experimental groups.
