# Supplementary material for: A graded neonatal mouse model of necrotizing enterocolitis demonstrates that mild enterocolitis is sufficient to activate microglia and increase cerebral cytokine expression
Source: PLoS One. 2025 May 30;20(5):e0323626. doi: 10.1371/journal.pone.0323626 (PMC12124527; doi:10.1371/journal.pone.0323626)
Supplement: S1 Table — P-values for the comparison between the survival curves of two experimental groups (indicated in the first row and column). A Kaplan-Meier survival analysis was used for mortality during feeding. Significant p-values (< 0.05) are in bold. (PDF) [file pone.0323626.s009.pdf]

Supporting Information

A graded neonatal mouse model of necrotizing enterocolitis demonstrates that mild enterocolitis is sufficient to activate microglia and increase cerebral cytokine expression

Sha, et al.

S1 Table. Comparisons of Kaplan-Meier survival curves (relates to Fig 1A).

	0% DSS	0.25% DSS	1% DSS	2% DSS	3% DSS
0% DSS					
0.25% DSS	*0.30*				
1% DSS	*0.73*	*0.23*			
2% DSS	***<0.0001***	***0.0054***	***<0.0001***		
3% DSS	***<0.0001***	***0.0011***	***<0.0001***	***0.0088***	

P-values for the comparison between the survival curves of two experimental groups (indicated in the first row and column). A Kaplan-Meier survival analysis was used for mortality during feeding. Significant *p-values* (< 0.05) are in ***bold***.
