# Supplementary material for: A graded neonatal mouse model of necrotizing enterocolitis demonstrates that mild enterocolitis is sufficient to activate microglia and increase cerebral cytokine expression
Source: PLoS One. 2025 May 30;20(5):e0323626. doi: 10.1371/journal.pone.0323626 (PMC12124527; doi:10.1371/journal.pone.0323626)
Supplement: S2 Table — Mean weights, standard error of mean (SEM), and counts of all mice that survived the entire feeding protocol. (PDF) [file pone.0323626.s010.pdf]

Supporting Information

A graded neonatal mouse model of necrotizing enterocolitis demonstrates that mild enterocolitis is sufficient to activate microglia and increase cerebral cytokine expression

Sha, et al.

S2 Table. Normalized weights of mice that survived to the final 72-hour timepoint (relates to Fig 1B).

	**Normalized Weight**		
**Experimental Group**	Mean	SEM	**N (mice)**
0% DSS	1.13	0.01	20
0.25% DSS	1.14	0.02	11
1% DSS	1.11	0.02	13

Mean weights, standard error of mean (SEM), and counts of all mice that survived the entire feeding protocol.
