# Supplementary material for: A graded neonatal mouse model of necrotizing enterocolitis demonstrates that mild enterocolitis is sufficient to activate microglia and increase cerebral cytokine expression
Source: PLoS One. 2025 May 30;20(5):e0323626. doi: 10.1371/journal.pone.0323626 (PMC12124527; doi:10.1371/journal.pone.0323626)
Supplement: S3 Table — P-values for the comparison between weight gain curves of two experimental groups (indicated in the first row and column). A two-way analysis of variance (ANOVA) with Tukey’s post-hoc was used for statistical analysis of weight gain curves. Significant p-values (< 0.05) are in bold. (PDF) [file pone.0323626.s011.pdf]

## Supporting Information

A graded neonatal mouse model of necrotizing enterocolitis demonstrates that mild enterocolitis is sufficient to activate microglia and increase cerebral cytokine expression

Sha, et al.

**S3 Table. Comparisons of normalized weight curves during feeding (relates to Fig 1B).**

| | 0% DSS | 0.25% DSS | 1% DSS | 2% DSS | 3% DSS |
|---|---|---|---|---|---|
| 0% DSS | | | | | |
| 0.25% DSS | *0.18* | | | | |
| 1% DSS | *0.47* | *0.98* | | | |
| 2% DSS | *0.56* | ***0.031*** | *0.087* | | |
| 3% DSS | ***0.043*** | ***0.0020*** | ***0.0052*** | *0.48* | |

*P-values* for the comparison between weight gain curves of two experimental groups (indicated in the first row and column). A two-way analysis of variance (ANOVA) with Tukey's post-hoc was used for statistical analysis of weight gain curves. Significant *p-values* (< 0.05) are in ***bold***.
