# Supplementary material for: A graded neonatal mouse model of necrotizing enterocolitis demonstrates that mild enterocolitis is sufficient to activate microglia and increase cerebral cytokine expression
Source: PLoS One. 2025 May 30;20(5):e0323626. doi: 10.1371/journal.pone.0323626 (PMC12124527; doi:10.1371/journal.pone.0323626)
Supplement: S4 Table — Mean ± SEM for the behavior score at each feeding time (in hours); the values in the row below are the number of mice alive during that feeding time for behavioral assessment. CSS measures were determined using a scoring system from Zani et al. (2008) [18]. (PDF) [file pone.0323626.s012.pdf]

Supporting Information

A graded neonatal mouse model of necrotizing enterocolitis demonstrates that mild enterocolitis is sufficient to activate microglia and increase cerebral cytokine expression

Sha, et al.

S4 Table. Clinical sickness scores (CSS) of mice during feeding (relates to Fig 1C).

	Clinical Sickness Score				
**Exp Group**	12	24	36	48	60
0% DSS	0 ± 0	0 ± 0	0.07 ± 0.05	1.48 ± 0.26	1.45 ± 0.28
N (mice)	29	29	29	23	20
0.25% DSS	0 ± 0	0 ± 0	0.05 ± 0.05	1.05 ± 0.22	0.62 ± 0.31
N (mice)	24	24	21	19	13
1% DSS	0 ± 0	0 ± 0	0.38 ± 0.11	1.55 ± 0.27	1.87 ± 0.27
N (mice)	23	22	21	20	15
2% DSS	0.29 ± 0.18	0.29 ± 0.18	1.40 ± 0.40	2.33 ± 0.33	3.00 ± 1.00
N (mice)	7	7	5	3	2

Mean ± SEM for the behavior score at each feeding time (in hours); the values in the row below are the number of mice alive during that feeding time for behavioral assessment. CSS measures were determined using a scoring system from Zani et al. (2008) [2].

2. Zani A, Cordischi L, Cananzi M, De Coppi P, Smith VV, Eaton S, Pierro A: **Assessment of a neonatal rat model of necrotizing enterocolitis.** *Eur J Pediatr Surg* 2008, **18:**423-426.
