# Supplementary material for: A graded neonatal mouse model of necrotizing enterocolitis demonstrates that mild enterocolitis is sufficient to activate microglia and increase cerebral cytokine expression
Source: PLoS One. 2025 May 30;20(5):e0323626. doi: 10.1371/journal.pone.0323626 (PMC12124527; doi:10.1371/journal.pone.0323626)
Supplement: S5 Table — P-values for the comparison of behavioral scores between two groups at each feeding time (in hours). A two-way ANOVA with Tukey’s post-hoc test was used for statistical analysis of the behavioral scores during feeding. Significant p-values (< 0.05) are in bold. (PDF) [file pone.0323626.s013.pdf]

Supporting Information

A graded neonatal mouse model of necrotizing enterocolitis demonstrates that mild enterocolitis is sufficient to activate microglia and increase cerebral cytokine expression

Sha, et al.

S5 Table. Comparisons of CSS during feeding (relates to Fig 1C).

	Feeding Time (hours)					
**Comparison**	12	24	36	48	60	**Overall**
0% vs 0.25% DSS	*>0.99*	*>0.99*	*>0.99*	*0.18*	***0.0034***	***0.028***
0% vs 1% DSS	*>0.99*	*>0.99*	*0.38*	*0.99*	*0.27*	*0.29*
0% vs 2% DSS	*0.75*	*0.75*	***0.0004***	*0.17*	***0.012***	***<0.0001***
0.25% vs 1% DSS	*>0.99*	*>0.99*	*0.38*	*0.10*	***<0.0001***	***0.0001***
0.25% vs 2% DSS	*0.76*	*0.76*	***0.0004***	***0.013***	***<0.0001***	***<0.0001***
1% vs 2% DSS	*0.76*	*0.76*	***0.014***	*0.24*	*0.12*	***0.0003***

P-values for the comparison of behavioral score between two groups at each feeding time (in hours). A two-way ANOVA with Tukey's post-hoc test was used for statistical analysis of the behavioral scores during feeding. Significant *p-values* (< 0.05) are in ***bold***.
