# Supplementary material for: A graded neonatal mouse model of necrotizing enterocolitis demonstrates that mild enterocolitis is sufficient to activate microglia and increase cerebral cytokine expression
Source: PLoS One. 2025 May 30;20(5):e0323626. doi: 10.1371/journal.pone.0323626 (PMC12124527; doi:10.1371/journal.pone.0323626)
Supplement: S6 Table — Mean external bowel scores, SEM, and counts of mice whose intestines were evaluated macroscopically. Bowels were evaluated using a scoring system from Zani et al. (2008) [18]. (PDF) [file pone.0323626.s014.pdf]

## Supporting Information

A graded neonatal mouse model of necrotizing enterocolitis demonstrates that mild enterocolitis is sufficient to activate microglia and increase cerebral cytokine expression
Sha, et al.

**S6 Table. External bowel scores of control and DSS-fed mice (relates to Fig 2B).**

| | External Bowel Score | | |
|---|---|---|---|
| **Experimental Group** | Mean | Standard Error of Mean | **N (intestines)** |
| 0% DSS | 0.6 | 0.2 | 18 |
| 0.25% DSS | 1.2 | 0.2 | 13 |
| 1% DSS | 4.0 | 0.3 | 18 |
| 2% DSS | 4.7 | 0.4 | 7 |

Mean external bowel scores, SEM, and counts of mice whose intestines were evaluated macroscopically. Bowels were evaluated using a scoring system from Zani et al. (2008) [2].


2. Zani A, Cordischi L, Cananzi M, De Coppi P, Smith VV, Eaton S, Pierro A: **Assessment of a neonatal rat model of necrotizing enterocolitis.** *Eur J Pediatr Surg* 2008, **18:**423-426.
