# Supplementary material for: A graded neonatal mouse model of necrotizing enterocolitis demonstrates that mild enterocolitis is sufficient to activate microglia and increase cerebral cytokine expression
Source: PLoS One. 2025 May 30;20(5):e0323626. doi: 10.1371/journal.pone.0323626 (PMC12124527; doi:10.1371/journal.pone.0323626)
Supplement: S7 Table — P-values for the comparison of external bowel scores between two groups (indicated in the first row and column). A one-way ANOVA with Tukey’s post-hoc test was used for statistical analysis of the external bowel scores. Significant p-values (< 0.05) are emphasized in bold. (PDF) [file pone.0323626.s015.pdf]

Supporting Information

A graded neonatal mouse model of necrotizing enterocolitis demonstrates that mild enterocolitis is sufficient to activate microglia and increase cerebral cytokine expression

Sha, et al.

S7 Table. Comparisons of external bowel scores. (relates to Fig 2B).

	0% DSS	0.25% DSS	1% DSS	2% DSS
0% DSS				
0.25% DSS	*0.33*			
1% DSS	***<0.0001***	***<0.0001***		
2% DSS	***<0.0001***	***<0.0001***	*0.27*	

P-values for the comparison of external bowel score between two groups (indicated in the first row and column). A one-way ANOVA with Tukey's post-hoc test was used for statistical analysis of the external bowel scores. Significant *p-values* (< 0.05) are emphasized in ***bold***.
