# Supplementary material for: A graded neonatal mouse model of necrotizing enterocolitis demonstrates that mild enterocolitis is sufficient to activate microglia and increase cerebral cytokine expression
Source: PLoS One. 2025 May 30;20(5):e0323626. doi: 10.1371/journal.pone.0323626 (PMC12124527; doi:10.1371/journal.pone.0323626)
Supplement: S8 Table — P-values for the comparison of intestinal pathology scores between two groups at each region of the GI tract. A two-way ANOVA with Tukey’s post-hoc test was used for statistical analysis of the intestinal pathology scores. Significant p-values (< 0.05) are in bold. (PDF) [file pone.0323626.s016.pdf]

Supporting Information

A graded neonatal mouse model of necrotizing enterocolitis demonstrates that mild enterocolitis is sufficient to activate microglia and increase cerebral cytokine expression

Sha, et al.

S8 Table. Comparisons of intestinal pathology scores across bowel regions (relates to Fig 2C).

**Comparison**	**P-value**
Proximal Small Bowel vs. Distal Small Bowel	*0.98*
Proximal Small Bowel vs. Colon	***0.0004***
Distal Small Bowel vs. Colon	***0.0005***

P-values for the comparison of intestinal pathology scores between two groups at each region of the GI tract. A two-way ANOVA with Tukey's post-hoc test was used for statistical analysis of the intestinal pathology scores. Significant *p-values* (< 0.05) are in ***bold***.
