# Supplementary material for: A graded neonatal mouse model of necrotizing enterocolitis demonstrates that mild enterocolitis is sufficient to activate microglia and increase cerebral cytokine expression
Source: PLoS One. 2025 May 30;20(5):e0323626. doi: 10.1371/journal.pone.0323626 (PMC12124527; doi:10.1371/journal.pone.0323626)
Supplement: S10 Table — Mean Ki-67+ cell count, SEM, and number of all intestinal crypts counted. (PDF) [file pone.0323626.s018.pdf]

Supporting Information

A graded neonatal mouse model of necrotizing enterocolitis demonstrates that mild enterocolitis is sufficient to activate microglia and increase cerebral cytokine expression

Sha, et al.

S10 Table. Ki-67+ cell counts per small intestinal crypt in mice (relates to Fig 2D).

	**Ki-67+ Cell Counts per Crypt**		
**Experimental Group**	Mean	SEM	**N (crypts)**
0% DSS	13.2	0.9	27
0.25% DSS	14.7	0.8	42
1% DSS	13.2	1.0	16
2% DSS	10.9	1.1	7

Mean Ki-67+ cell count, SEM, and number of all intestinal crypts counted.
