# Supplementary material for: A graded neonatal mouse model of necrotizing enterocolitis demonstrates that mild enterocolitis is sufficient to activate microglia and increase cerebral cytokine expression
Source: PLoS One. 2025 May 30;20(5):e0323626. doi: 10.1371/journal.pone.0323626 (PMC12124527; doi:10.1371/journal.pone.0323626)
Supplement: S11 Table — P-values for the comparison of Ki-67+ small intestinal crypt counts between two groups (indicated in the first row and column). A one-way ANOVA with Tukey’s post-hoc test was used for statistical analysis of the Ki-67+ counts. Significant p-values (< 0.05) are in bold. (PDF) [file pone.0323626.s019.pdf]

Supporting Information

A graded neonatal mouse model of necrotizing enterocolitis demonstrates that mild enterocolitis is sufficient to activate microglia and increase cerebral cytokine expression
Sha, et al.

S11 Table. Comparisons of Ki-67+ scores (relates to Fig 2D).

	0% DSS	0.25% DSS	1% DSS	2% DSS
0% DSS				
0.25% DSS	*0.64*			
1% DSS	*0.96*	*0.40*		
2% DSS	***0.019***	***0.0008***	*0.091*	

P-values for the comparison of Ki-67+ small intestinal crypt counts between two groups (indicated in the first row and column). A one-way ANOVA with Tukey's post-hoc test was used for statistical analysis of the Ki-67+ counts. Significant *p-values* (< 0.05) are in ***bold***.
