# Supplementary material for: A graded neonatal mouse model of necrotizing enterocolitis demonstrates that mild enterocolitis is sufficient to activate microglia and increase cerebral cytokine expression
Source: PLoS One. 2025 May 30;20(5):e0323626. doi: 10.1371/journal.pone.0323626 (PMC12124527; doi:10.1371/journal.pone.0323626)
Supplement: S12 Table — P-values for the comparison between two groups (indicated in the first row and column) of the proportion of neurons among all cells in the CA1 hippocampal region. A one-way ANOVA with Tukey’s post-hoc test was used for statistical analysis of the neuron proportions. Significant p-values (< 0.05) are in bold. (PDF) [file pone.0323626.s020.pdf]

Supporting Information

A graded neonatal mouse model of necrotizing enterocolitis demonstrates that mild enterocolitis is sufficient to activate microglia and increase cerebral cytokine expression

Sha, et al.

S12 Table. Comparisons of neuron proportions in CA1 hippocampus (relates to S8B Fig).

	0% DSS	0.25% DSS	1% DSS	2% DSS
0% DSS				
0.25% DSS	*>0.99*			
1% DSS	*0.49*	*0.63*		
2% DSS	*0.43*	*0.31*	***0.027***	

P-values for the comparison between two groups (indicated in the first row and column) of the proportion of neurons among all cells in the CA1 hippocampal region. A one-way ANOVA with Tukey's post-hoc test was used for statistical analysis of the neuron proportions. Significant *p-values* (< 0.05) are in ***bold***.
