# Supplementary material for: A graded neonatal mouse model of necrotizing enterocolitis demonstrates that mild enterocolitis is sufficient to activate microglia and increase cerebral cytokine expression
Source: PLoS One. 2025 May 30;20(5):e0323626. doi: 10.1371/journal.pone.0323626 (PMC12124527; doi:10.1371/journal.pone.0323626)
Supplement: S13 Table — P-values for the comparison between two groups (indicated in the first row and column) of the proportion of microglia among all cells in the CA1 hippocampal region. A one-way ANOVA with Tukey’s post-hoc test was used for statistical analysis of the microglia proportions. Significant p-values (< 0.05) are in bold. (PDF) [file pone.0323626.s021.pdf]

Supporting Information

A graded neonatal mouse model of necrotizing enterocolitis demonstrates that mild enterocolitis is sufficient to activate microglia and increase cerebral cytokine expression

Sha, et al.

S13 Table. Comparisons of microglia proportions in CA1 hippocampus (relates to S8C Fig).

	0% DSS	0.25% DSS	1% DSS	2% DSS
0% DSS				
0.25% DSS	*0.50*			
1% DSS	*0.33*	***0.018***		
2% DSS	*0.66*	*0.068*	*0.94*	

P-values for the comparison between two groups (indicated in the first row and column) of the proportion of microglia among all cells in the CA1 hippocampal region. A one-way ANOVA with Tukey's post-hoc test was used for statistical analysis of the microglia proportions. Significant *p-values* (< 0.05) are in ***bold***.
