# Supplementary material for: A graded neonatal mouse model of necrotizing enterocolitis demonstrates that mild enterocolitis is sufficient to activate microglia and increase cerebral cytokine expression
Source: PLoS One. 2025 May 30;20(5):e0323626. doi: 10.1371/journal.pone.0323626 (PMC12124527; doi:10.1371/journal.pone.0323626)
Supplement: S14 Table — Sholl analysis was used to quantify dendritic branching complexity of microglia, with a set distance interval of 5 microns (minimum: 5 microns; maximum: 70 microns). Values are the p-values for the comparison of dendritic branching points between two groups at each distance from the cell soma (microns). A two-way analysis of variance (ANOVA) with Tukey’s post-hoc test was used for statistical analysis of the Sholl data, which is graphically summarized in Fig 7. Significant p-values (< 0.05) are in bold. (PDF) [file pone.0323626.s022.pdf]

Supporting Information

A graded neonatal mouse model of necrotizing enterocolitis demonstrates that mild enterocolitis is sufficient to activate microglia and increase cerebral cytokine expression
Sha, et al.

S14 Table. Sholl analysis comparisons at distances beyond 40 microns from microglial cell soma (relates to Fig 7 and Table 3).

	**Distance from Cell Soma (microns)**						
**Comparison**	45	50	55	60	65	70	Overall
0% vs 0.25% DSS	*0.92*	*0.88*	*0.96*	*>0.99*	*>0.99*	*>0.99*	***<0.0001***
0% vs 1% DSS	*0.81*	*0.86*	*0.96*	*>0.99*	*>0.99*	*>0.99*	***<0.0001***
0% vs 2% DSS	*0.85*	*0.89*	*0.97*	*>0.99*	*>0.99*	*>0.99*	***<0.0001***
0.25% vs 1% DSS	*0.99*	*>0.99*	*>0.99*	*>0.99*	*>0.99*	*>0.99*	*0.089*
0.25% vs 2% DSS	*>0.99*	*>0.99*	*>0.99*	*>0.99*	*>0.99*	*>0.99*	*0.97*
1% vs 2% DSS	*>0.99*	*>0.99*	*>0.99*	*>0.99*	*>0.99*	*>0.99*	***0.020***

Sholl analysis was used to quantify dendritic branching complexity of microglia, with a set distance interval of 5 microns (minimum: 5 microns; maximum: 70 microns). Values are the *p-values* for the comparison of dendritic branching points between two groups at each distance from the cell soma (microns). A two-way analysis of variance (ANOVA) with Tukey's post-hoc test was used for statistical analysis of the Sholl data, which is graphically summarized in Fig 7. Significant *p-values* (< 0.05) are in ***bold***.
